# Identification of 1,3-1,4-β-D-glucanase (OsDLH) genes and analysis of haplotype diversity in rice

**DOI:** 10.3389/fpls.2025.1690795

**Published:** 2025-10-16

**Authors:** Manqiong Zhu, You Zhou, Meng Zhang, Yaling Bao, Chunni Wang, Wenhao Lv, Chenghang Tang, Jun Zhu, Kailiang Tao, Ruofan Chen, Jinguo Zhang, Ergashev Mukhammadjon Arabboevich, Yingyao Shi

**Affiliations:** ^1^ School of Agronomy, Anhui Agricultural University, Hefei, China; ^2^ Rice Reserach Institute of Uzbekistan, Sholikor, Tashkent Region, Uzbekistan

**Keywords:** rice, 1,3-1,4-β-D-glucanase, haplotype, abiotic stress, genetic diversity

## Abstract

1,3-1,4-β-D-glucanase (*DLH*) is a fibrolytic enzyme playing important roles in plant growth, development, and stress response. In this study, we identified 11 *DLH* genes in *japonica* rice (Nipponbare), and analyzed their chromosomal localization, physicochemical properties, subcellular localization, evolutionary relationships, and collinearity. We also performed *cis*-acting element identification, gene expression profiling, and qPCR verification under abiotic stress, as well as conducted diversity analysis of gene-CDS-haplotypes (gcHaps) in 3,010 rice germplasms to dissect the potential functions of these *OsDLH* genes. The results showed that *OsDLH1, OsDLH5*, and *OsDLH10* can be utilized to improve the tolerance of rice to abiotic stress. *OsDLH1*, *OsDLH2* and *OsDLH8* are highly expressed in roots. Under high-salt stress, the expression of *OsDLH6* and *OsDLH10* in stems and leaves increased. These results indicated that *OsDLH1* and *OsDLH5* have specific expression patterns in response to different environmental signals, implying that they may play different roles in the growth and development process of rice and the stress response mechanism. Haplotype analysis indicated population differentiation of the Os*DLH* gene family in rice. The major gcHaps at most *OsDLH* loci are significantly associated with yield traits. Some genes of this family have potential application value in improving stress resistance and yield traits of rice.

## 1 Introduction

Rice (*Oryza sativa* L.) is one of the most important edible crops only next to wheat worldwide (Khaskhali et al., 2025). However, rice is constantly confronted with the threats from various stresses (Ramasamy et al., 2025), especially drought stress, which poses serious threats to global food security (Sagar et al., 2025). Heat and cold stresses also cause significant adversities during rice growth. Heat stress has particularly negative impacts on rice production. Hence, it is of vital importance to understand the physiological basis underlying heat tolerance in rice for better addressing the negative impacts of heat stress (Beena et al., 2025). Rice is also highly sensitive to low temperatures, and therefore cold stress is also a significant factor limiting its growth, especially during the germination stage (Jo et al., 2025). Moreover, high salinity is another major abiotic stress with substantial adverse impacts on the growth and production of rice (Zhao et al., 2025). These adverse effects of various stresses highlight the necessity of analyzing its complex genetic structure (Lee et al., 2025).

The plant endo-β-1,4 glucanases hydrolyze polysaccharides with a β-1,4 glucan backbone, participate in cellulose biosynthesis, and play important roles in cell wall biosynthesis and remodeling and fruit abscission (He et al., 2018; Li et al., 2019).1,3-1,4-β-glucanase (*DLH*) and 1,3-β-D-glucanase (laminarinase) are fibrolytic enzymes playing important roles in the hydrolysis of polysaccharide components (Liu et al., 2012). *DLH* is present in a variety of bacteria, fungi, plants, and animals (Chaari and Chaabouni, 2019). In plants, *DLH* is widely distributed in the cell walls of gramineous plants and some other vascular plants (Chang et al., 2023), serving as a polysaccharide component of the cell walls. It is particularly abundant in the endosperm cell walls of commercially valuable cereals such as barley, rye, sorghum, rice, and wheat (Planas, 2000), and can hydrolyze the glycosidic bonds with mixed linkages in glucan (Tsai et al., 2003). The glucan content of rice is a key factor defining its nutritional and economic value. Starch and its derivatives have many industrial applications such as in fuel and material production. Non-starch glucans such as (1,3;1,4)-beta-D-glucan (mixed-linkage beta-glucan, MLG) have many benefits to human health, including lowering cholesterol, boosting the immune system, and modulating the gut microbiome (Panahabadi et al., 2021). Exo- and endo-glucanases mediate the specific degradation of cell wall *DLH*, and are associated with auxin-mediated growth and development of cereal coleoptiles (Inouhe et al., 2000). To date, *DLH* has also been studied in sorghum (Demirbas, 2005). It is well known that germination has a highly significant impact on enzyme activity (Uriyo and Eigel, 2000). In the study of the stress resistance of *DLH*, the enzyme exhibited high continuous synthesis capacity of regenerated amorphous cellulose and could maintain over 90% of its activity at 80 °C for a long time, indicating excellent thermal stability of its enzymatic activity (Hussain et al., 2025). In the rice genome, endo-1,4-β-D-glucanase forms a multigene family, with each member showing different expression patterns in various organs. These results suggest that endo-1,4-β-D-glucanase may play multiple roles in the growth and development of rice plants (Zhou et al., 2006). *DLH* and laminarinase may be related to lodging resistance. Some studies have demonstrated that the expression of the *Gns1* gene increased sharply in the stem, and this gene may encode *DLH*, which catalyzes the degradation of (1-3,1-4)-β-glucan. Baba et al. studied the decomposition mechanism of (1-3,1-4)-β-glucan in rice stems and its possible association with lodging resistance (Baba et al., 2001).

The sequencing of 3,010 rice germplasms (3KRG) from 89 countries worldwide has been completed, which provides a wealth of genetic information about rice (Wang et al., 2018). The 3KRG is a gigabyte-scale dataset that encompasses the publicly available genomic sequences of more 3,000 rice germplasms and exhibits genetic and functional diversity (Li et al., 2014a). On the basis of 3KRG, haplotype data can be integrated to explore the functions of rice genes (Zeng et al., 2023; Cheng et al., 2023), which can not only reveal the diversity and complexity of gene families in rice, but also underscore the significance of haplotype data in deciphering gene functions and their relationships with various agronomic traits. With the progress of biomolecular research, there have been increasing studies of the expression, function, and resistance of *DLH* genes. However, there have been relatively few studies of the members in the rice *DLH* gene family, their evolutionary relationships, specific functions, genetic diversity, allelic variations, and associated agronomic traits. This is of particular importance for a comprehensive understanding of the functions and diversity of rice genes and gene networks as well as their associations with important agronomic traits to increase rice yield through some innovative breeding techniques and strategies.

In this study, we systematically identified the members of the rice *DLH* gene family, resulting in the identification of 11 *OsDLH* genes from the Nipponbare genome. Moreover, we investigated the gene structures, functions, expression patterns, and phylogenies, providing a foundation for further deciphering the functions, regulatory mechanisms, and utilization of the *OsDLH* gene family. Additionally, we analyzed the gene-CDS-haplotype (gcHap) diversity of *OsDLH* genes and their associations with various agronomic traits, providing a theoretical basis for future novel breeding technologies.

## 2 Materials and methods

### 2.1 Identification of members in Rice *DLH* family

To identify the *DLH* family genes in rice, we accessed the Ensembl Plants database (https://plants.ensembl.org/index.html) and downloaded the genomes of *Oryza sativa japonica* (Nipponbare IRGSP-1.0), *Triticum aestivum* (IWGSC), *Arabidopsis thaliana* (TAIR10), *Hordeum vulgare* (MorexV3_pseudomolecules_assembly), *Sorghum bicolor* (Sorghum bicolor NCBIV3), and *Zea mays* (Zm-B73-REFERENCE-NAM-5.0) on April 7, 2025, which were denoted as Os, Ta, At, Hv, Sb, and Zm, respectively. The hidden Markov model (HMM) of PF01738 was downloaded from the Pfam database (accessed on April 7, 2025; http://pfam.xfam.org/). Subsequently, the SimpleHmmSearch function of TBtools software was used to obtain potential *DLH* gene and protein sequences. The NCBI website (https://www.ncbi.nlm.nih.gov/Structure/bwrpsb/bwrpsb.cgi) was utilized to analyze the genetic domains. A comprehensive analysis was carried out by combining functional annotations for further screening to identify the family members, which were then renamed according to their positions on the chromosomes. The online tool Cell-PLoc2.0 (http://www.csbio.sjtu.edu.cn/bioinf/plant-multi/) was used for subcellular localization prediction (Chou and Shen, 2008). In addition, the ProteinParamterCalc function of TBtools software was used to analyze the chemical properties such as isoelectric point (pI) and molecular weight (Da) of the *DLH* genes in rice (Chen et al., 2020).

### 2.2 Evolutionary analysis of *DLH* genes

To investigate the evolutionary relationships of *DLH* genes, we collected *DLH* genes from rice, wheat, foxtail millet, barley, and maize. A phylogenetic tree was constructed using the Neighbor-joining method in MEGA11 software. The online drawing website iTOL (accessed on April 7, 2025; https://itol.embl.de/) was used to beautify the phylogenetic tree (Letunic and Bork, 2016). More detailed procedures have been described in a previous study (Rui et al., 2022).

### 2.3 Analysis of *cis-acting elements*, conserved motifs, and conserved domains of *DLH* proteins

The gene structure annotation file was downloaded from the EnsemblPlants website, and the Gene Structure View (Advanced) function in TBtools software was used for visualization (Chen et al., 2020). The motifs were obtained using MEME (https://meme-suite.org/meme/tools/meme; April 7, 2025) and the Simple MEME Wrapper function of TBtools software (Bailey et al., 2015). The conserved *DLH* protein sequences were analyzed with the number of motifs being set to 8. The conserved domains were analyzed through the NCBI search function and visualized by Tbtools. The *cis-acting elements* were analyzed using the Gff3 sequence extraction function in TBtools to extract the 2000-bp region upstream of the CDS and the promoter sequences of the *DLH* genes. The obtained results were submitted to the PlantCARE database (http://bioinformatics.psb.ugent.be/webtools/plantcare/html/access) for analyzing the promoter region (Lescot et al., 2002). The results were filtered according to the information in the table, retaining the files for review, and then the SimpleBioSequenceViewer function of TBtools software was used for visualization (Chen et al., 2020).

### 2.4 Collinearity analysis of *DLH* genes in rice

First, the GFF files and DNA files of the target genomes were downloaded from the EnsemblPlants website. Then, TBtools was used to obtain the gene-pair files between rice and other plant species such as Gossypium raimondii, Arabidopsis thaliana, Solanum lycopersicum, Cucumis sativus, Citrullus lanatus, Glycine max, Zea mays, Setaria italica, Hordeum vulgare, and Triticum aestivum. The one-step MCScanX and AdvanceCircos functions of TBtools were used to analyze the collinearity of duplicated gene pairs in ten different species (Nipponbare, Gr, At, Sl, Cs, Cl, Gm, Hv, Ta, Zm, and Si). Finally, the collinearity results were visualized using the chromosome length files and genome comparison files.

### 2.5 RNA-seq-based gene expression profiling

From the RNA-seq database (PPRD, http://ipf.sustech.edu.cn/pub/plantrna/; accessed on April 7, 2025), the RNA-seq expression data of rice *DLH* genes (presented as FPKM values, Fragments Per Kilobase of exon model per Million mapped reads) were retrieved (Yu et al., 2022). For data from multiple tissue sites and different developmental stages, and under abiotic stress conditions, the average FPKM values from multiple libraries were used for analysis. The GraphPad Prism 8.0.2 software was used to standardize the data.The Tbtools software was used to create heatmaps for visual representation of the expression patterns (Chen et al., 2020).

### 2.6 Material treatment

In early 2025, at the Crop Molecular Breeding Innovation Center of Anhui Agricultural University, the rice seeds (Nipponbare) were disinfected with 3% sodium hypochlorite for 30 min, germinated at 28°C for 3 days, and then transplanted into hydroponic boxes filled with Hoagland nutrient solution. The plants were placed in an intelligent light-temperature incubator under normal conditions (28°C for 12 h during the day, 28°C for 12 h at night, 70% humidity, and light intensity of 20,000 lux). The seedlings were treated with low temperature (4°C) and high temperature (42°C), respectively. Rice leaves were collected at 0, 1, 3, 6, 12, and 24 h, immediately placed in liquid nitrogen, and stored at –80°C for total RNA extraction.

### 2.7 Quantitative real-time PCR analysis

The collected samples were ground in liquid nitrogen using a mortar and pestle. The TaKaRa MiniBEST Plant RNA Extraction Kit (TaKaRa, Japan) was employed to extract RNA. The obtained cDNA was reverse transcribed using the TAKARA Reverse Transcription Kit (TAKARA, Japan). Gene expression was analyzed via qRT-PCR. Primers for the selected *OsDLH*1 and *OsDLH*5 were designed (**Supplementary Table S7**). The relative quantitative expression was normalized to the reference gene OsActin1 (LOC-Os03g61970) (Cui et al., 2020). Real-time fluorescence quantitative detection was performed using a LightCyler96 quantitative PCR instrument. The amplification system had a volume of 20 μL, with 2 μL of cDNA, 0.8 μL each of forward and reverse primers, 10 μL of AceQ Universal SYBR qPCR Premix, and 6.4 μL of ddH_2_O. The program was set as pre-denaturation at 95°C for 5 min; denaturation at 95°C for 10 s, annealing and extension at 60°C for 30 s, for a total of 40 cycles. Sampling was conducted at six time points: 0, 1, 3, 6, 12, and 24 h, with three replicates for each time point. For each replicate, five rice seedlings were pooled for processing. In total, ninety rice seedlings were used for each treatment. Three biological replicates and three technical replicates were carried out. The 2^-ΔΔCt^ method was used to analyze the expression of each gene. The WPS2023 software was used for statistical analysis of the data, and the GraphPad Prism8 software was used for analysis of variance and graphing.

### 2.8 gcHap of *OsDLH* genes and gcHap diversity in modern and local rice varieties

Shannon’s equity (EH) and F-statistic was used to assess the gcHap diversity at the *OsDLH* loci in different rice populations. For each gene, Nei’s genetic diversity (INei) was estimated using gcHap data to measure the genetic differences between two populations and to quantify the genetic differentiation among populations (Castelli et al., 2019).The GraphPad Prism 8.0.2 software was used to standardize the data(INei). To understand how modern breeding has influenced the gcHap diversity of *OsDLH* genes in recent decades, we collected detailed information on a total of 3010 3KRG rice germplasms. Among them, 732 were identified as *indica* landraces (LANs-Xian), 328 as *japonica* landraces (LANs-Geng), 358 as modern *indica* varieties (MVs-Xian), and 139 as modern *japonica* varieties (MVs-Geng). First, on April 7, 2025, we downloaded the gcHap data of *OsDLH* genes from RFGB (accessed at RFGB Database (rmbreeding.cn)). Then, based on an R script, we calculated the drift frequency of the major gcHaps of each *OsDLH* gene between modern varieties (MVs) and landraces (LANs). Subsequently, we compared the gcHap distributions of modern Xian and Geng varieties with those of their respective landraces. Finally, the abovementioned data were plotted using GraphPad Prism 8 software.

### 2.9 Phenotypic determination of major gcHaps of *OsDLH* genes

First, we collected the phenotypic data of 15 agronomic traits from 3KRG, including days to heading (DTH, day), plant height (PH, cm), flag leaf length (FLL, cm), flag leaf width (FLW, cm), panicle number (PN, count), panicle length (PL, cm), culm number (CN, count), culm length (CL, cm), grain length (GL, mm), grain width (GW, mm), grain length-to-width ratio (GLWR, ratio), 1000-grain weight (TGW, g), leaf rolling index (LRI, %), seedling height (SH, cm), and lemma length (LL, mm). The phenotypic data of the 15 traits were downloaded from the RFGB website (accessed on April 7, 2025; RFGB Database (rmbreeding.cn)). Next, an R script was used to obtain the major gcHaps of all *OsDLH* genes. Finally, an R script was employed to correlate the major gcHaps with these agronomic traits in 3,010 rice germplasms. Significance was calculated using one-way analysis of variance, and the Tukey multiple-comparison method was used to compare the significance among the major gcHaps. The layout of the images was performed in Adobe Illustrator 2020 software.

### 2.10 Construction of the gcHap network of *OsDLH* genes

First, the gcHaps of *OsDLHs* were constructed using the pegas script in R (Paradis, 2010). The statistical parsimony algorithm was applied to generate a gcHap network for each *OsDLH* gene. This algorithm initially connects the most closely related haplotypes with the minimum number of mutations (Templeton et al., 1992). The layout of the images was completed using Adobe Illustrator 2023 software.

## 3 Results

### 3.1 Genome-wide identification and characterization of *OsDLH* genes

We identified 11 *OsDLH* genes in the Nipponbare genome. To further understand the characteristics of these genes, we analyzed their physicochemical properties, including sequence length, molecular weight (Da), isoelectric point (pI), instability index, aliphatic index, grand average of hydropathicity (GRAVY), and subcellular localization (**Supplementary Table S1**). The sequence lengths of these *OsDLH* proteins ranged from 159 to 290 amino acids (aa), the molecular weights from 17,307.84 Da (*OsDLH1*) to 31,582.32 Da (*OsDLH11*) with an average of approximately 25,852.04273 Da, and the pI values from 5.07 to 7.65 with an average of 6.05. Notably, nine out of the 11 *OsDLH* proteins (81.82%) had an instability index below 40, indicating that most of them are stable. In addition, the lowest aliphatic index was 78.32, demonstrating that *OsDLH* proteins have certain degrees of thermal stability. Six (54.55%) *OsDLH* proteins had negative GRAVY values, indicating that most *OsDLH* proteins are hydrophilic. Subcellular localization analysis showed that *OsDLH* proteins were localized to chloroplasts, suggesting that they may have important biological functions in chloroplasts.

### 3.2 Evolutionary relationships of *DLH* genes

**Figure 1:**
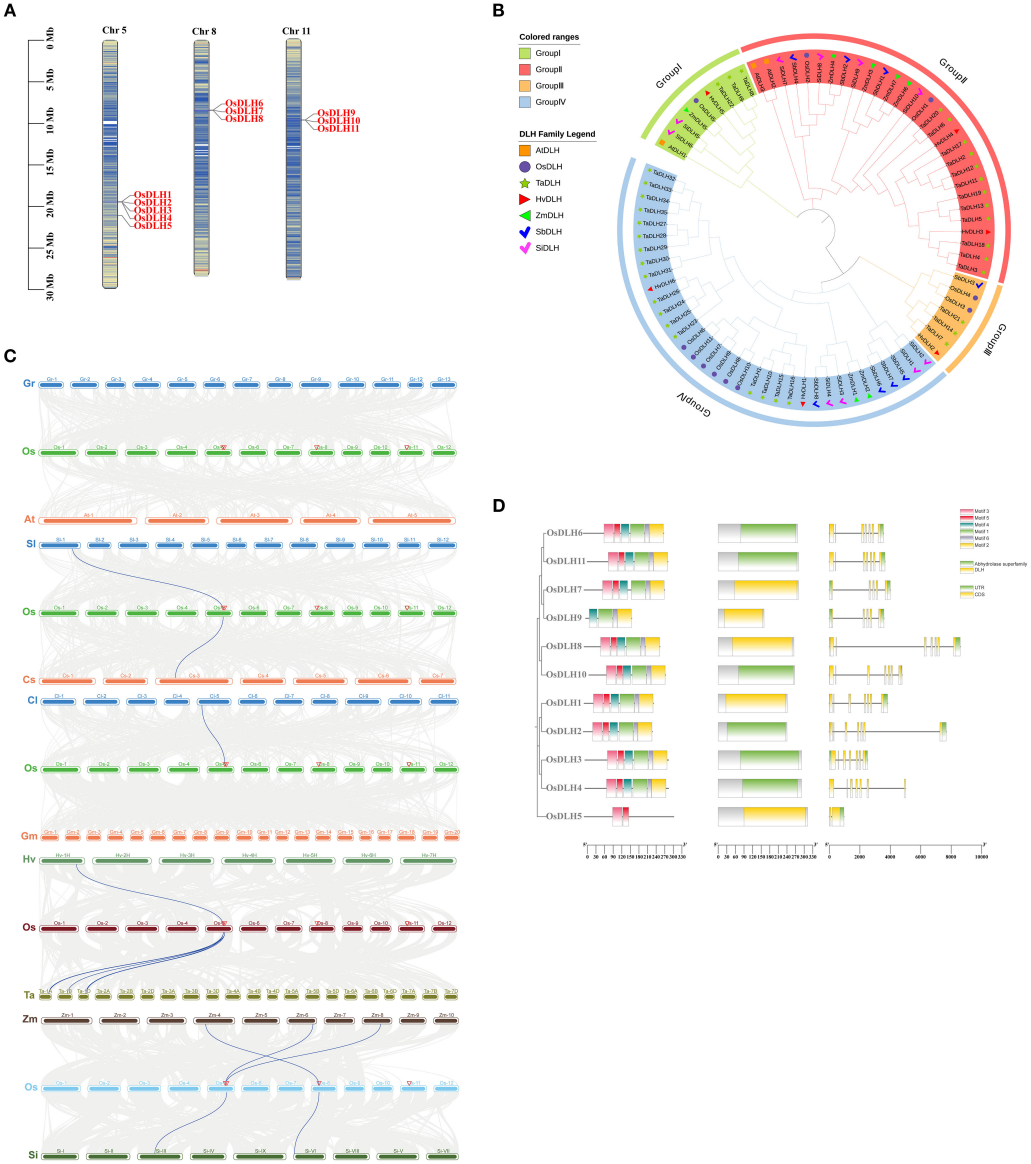
Characteristics of *OsDLH* genes. **(A)** Chromosomal localization of *OsDLHs*. **(B)** Phylogenetic tree of *DLH* genes from Nipponbare (Os), *Triticum aestivum* (Ta), *Arabidopsis thaliana* (At), *Hordeum vulgare* (Hv), *Sorghum bicolor* (Sb),Setaria italica(Si) and *Zea mays* (Zm). **(C)** Collinear relationships between *OsDLHs* and genes from other species. The collinear regions between the genome of Nipponbare and other species are represented by gray lines, and the collinear gene pairs are represented by blue lines. **(D)** Phylogenetic tree, motif prediction, domain, and exon-intron distribution of *OsDLHs* from left to right.

The chromosome localization map indicated that the 11 *OsDLH* genes are located on chromosomes 5, 8, and 11 of Nipponbare, respectively (**Figure 1A**). To further explore the evolutionary and structural characteristics of *DLH* genes, a phylogenetic tree was constructed, which included the *DLH* genes from Nipponbare, barley, Arabidopsis, wheat, sorghum, maize, and foxtail millet (**Supplementary Table S2**). These genes were classified into four major sub-families (I–IV) according to the proximity of their evolutionary relationships (**Figure 1B**). Group 1 contains 9 DLH genes, including 1 in rice, 1 in Arabidopsis, 2 in foxtail millet, 1 in maize, 3 in wheat, and 1 in barley.Group 2 contains 29 DLH genes, including 2 in rice, 2 in Arabidopsis, 4 in foxtail millet, 4 in maize, 12 in wheat, 2 in barley, and 3 in sorghum.Group 3 contains 7 DLH genes, including 2 in rice, 3 in wheat, 1 in barley, and 1 in sorghum.Group 4 contains 35 DLH genes, including 6 in rice, 4 in foxtail millet, 2 in maize, 17 in wheat, 2 in barley, and 4 in sorghum.Generally, the results of phylogenetic analysis not only present the phylogenetic relationships of DLH genes, but also provide some useful information for exploring the functions of unknown DLH genes in rice, Arabidopsis, barley, wheat, maize, sorghum and foxtail millet.

The collinearity relationship diagrams were respectively drawn between rice and Gossypium raimondii, Arabidopsis thaliana, tomato, cucumber, watermelon, soybean, barley, wheat, maize, and foxtail millet. The numbers of collinear gene pairs between rice and dicotyledonous plants such as Gossypium raimondii, Arabidopsis thaliana, tomato, cucumber, watermelon, and soybean were 0, 0, 1, 1, 1, and 0, respectively. While the numbers of collinear gene pairs between rice and monocotyledonous plants such as barley, wheat, maize, and foxtail millet were 1, 3, 3, and 2, respectively. This indicates that rice has more collinear genes with monocotyledonous plants than with dicotyledonous plants.This comparative analysis across different species helps to better understand the evolutionary dynamics of *DLH* family genes in various plants and their potential common functions in plant biology (Griffiths et al., 2006).

The motif prediction results (**Figure 1D**) showed that *OsDLHs* had highly similar motifs in both type and quantity. All proteins except for *OsDLH5* and *OsDLH9* contained motifs 1 to 6. The *OsDLH* genes also exhibited significant differences in gene length and exon number, and the differences were generally consistent with the analysis results of conserved motifs and conserved domains.

### 3.3 Analysis of *Cis*-acting elements of *OsDLH* genes

**Figure 2:**
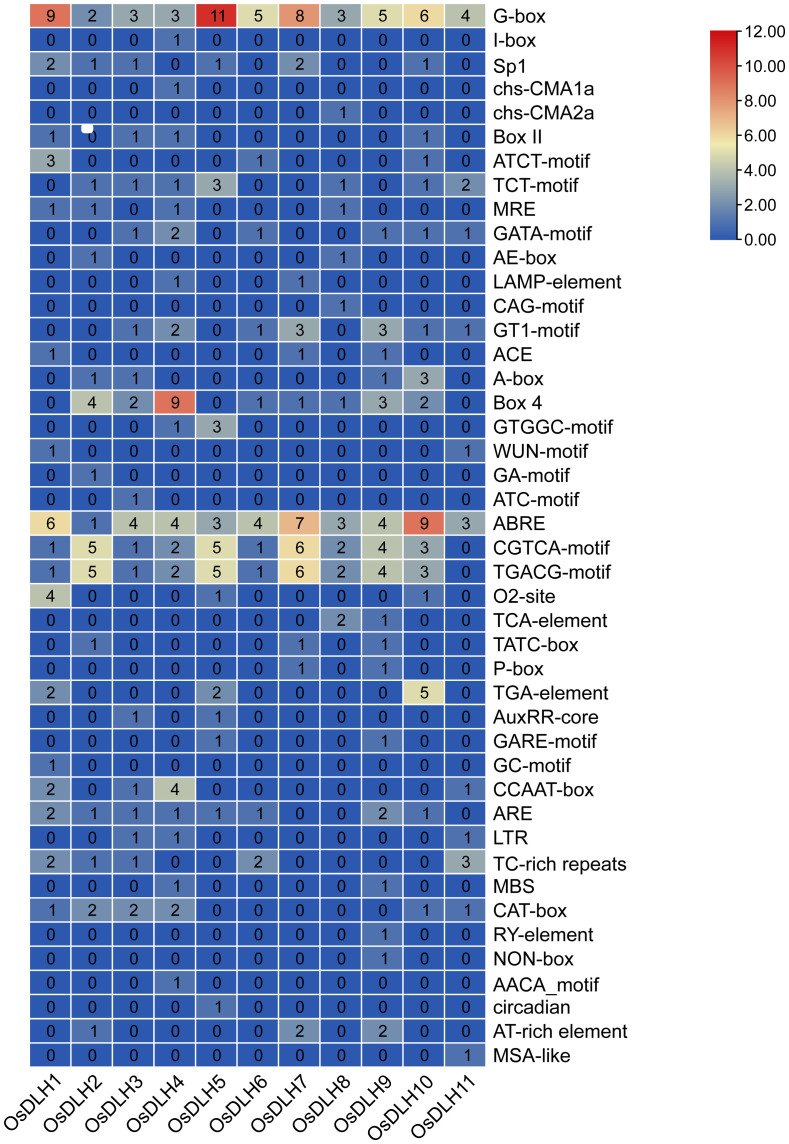
Analysis of *cis*-acting elements of *OsDLH* genes. Heatmap analysis of *cis*-acting elements in the promoter regions of *OsDLH* genes. In the heatmap, the values represent the numbers of different *cis*-acting elements, with a darker color indicating a larger number.

Analysis of the promoter regions of the 11 *OsDLH* genes identified a total of 45 types of *cis*-acting elements, and approximately 14.4% (68/472) of them were related to stress responses. Elements associated with methyl jasmonate (MeJA) and abscisic acid (ABA) responses were particularly prominent, accounting for 51.8% and 26.9% of the hormone-responsive elements, respectively. Additionally, elements related to light responses accounted for approximately 39% (188/472), while those related to plant growth were relatively scarce, only accounting for 4.8% (23/472) of the total. As shown in **Figure 2**, the heat-map analysis of the *cis*-acting elements in the promoter regions of the *OsDLH* genes revealed that elements such as G-box and ABRE were present in every member of the *OsDLH* gene family, indicating that this gene family may play an important role in plant responses to stresses and hormones through these *cis*-acting elements.

### 3.4 Expression profiles of *OsDLHs* in different tissues and under abiotic stresses

**Figure 3:**
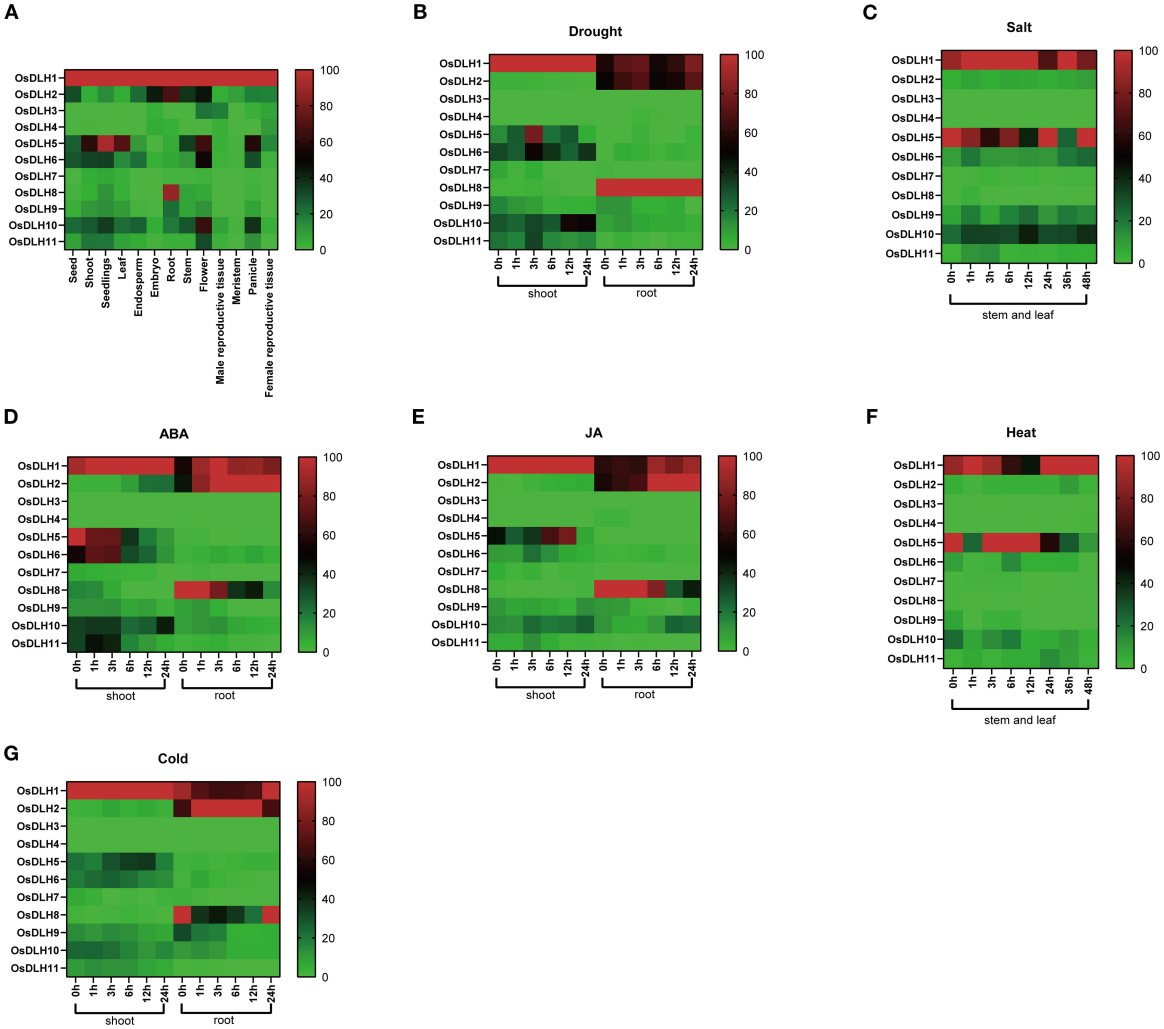
Expression analysis of *OsDLH* genes. The color coding represents changes in gene expression. Red indicates high expression, and blue indicates low expression. **(A)** Expression of *OsDLH* genes in leaves, roots, seedlings, stems, flowers, embryos, buds, meristems, male reproductive tissues, female reproductive tissues, panicles, and seeds. **(B)** Expression levels of *OsDLH* genes in roots and shoots after drought stress. **(C)** Expression levels of *OsDLH* genes in the stems and leaves of rice after high-salt stress. **(D)** Expression levels of *OsDLH* genes in roots and shoots after ABA hormone treatment. **(E)** Expression levels of *OsDLH* genes in roots and shoots after MeJA treatment. **(F)** Expression levels of *OsDLH* genes in stems and leaves after heat treatment. **(G)** Expression levels of *OsDLH* genes in roots and shoots after cold stress.

The *OsDLH* gene family displayed distinct tissue-specific expression patterns (**Figure 3A**). For instance, *OsDLH1, OsDLH5*, and *OsDLH10* were highly expressed in floral tissues and leaves, while *OsDLH1, OsDLH2*, and *OsDLH8* were highly expressed in roots. In addition, the expression patterns of *OsDLH* genes varied considerably under stress conditions. Under drought stress, the expression of *OsDLH5* in the aerial parts increased significantly at 0, 1, and 3 h, while that of *OsDLH7* in roots showed little change (**Figure 3B**). Under high-salt stress, the expression of *OsDLH6* and *OsDLH10* showed sharp increases in stems and leaves (**Figure 3C**). Moreover, ABA and MeJA treatments led to increases in the expression level of *OsDLH10* in roots and shoots (**Figure 3D, E**), whereas nearly no expression of *OsDLH3* and *OsDLH4*. After heat treatment, the expression levels of *OsDLH5* and *OsDLH6* in stems and leaves decreased (**Figure 3F**), while under cold stress, the expression of *OsDLH1* in shoots and *OsDLH2* in roots increased (**Figure 3G**). By integrating the results of the phylogenetic tree and expression patterns, it could be found that *OsDLH5* belonged to group 1. It had a relatively high expression level in roots under various stresses and hormone treatments, while its expression level in shoots was very low. *OsDLH1* and *OsDLH2* both belonged to group2. These two genes had relatively high expression levels in different parts under stress and hormone treatments. In contrast, *OsDLH3* and *OsDLH4* both belonged to group3, but were less expressed in different environments. These results demonstrated the expression patterns of *OsDLH* genes in different tissues and under various environmental stresses, offering crucial clues for further investigating the functions of these genes in rice growth, development, and stress responses.

### 3.5 Real-time fluorescent quantitative PCR analysis of *OsDLHs*

**Figure 4:**
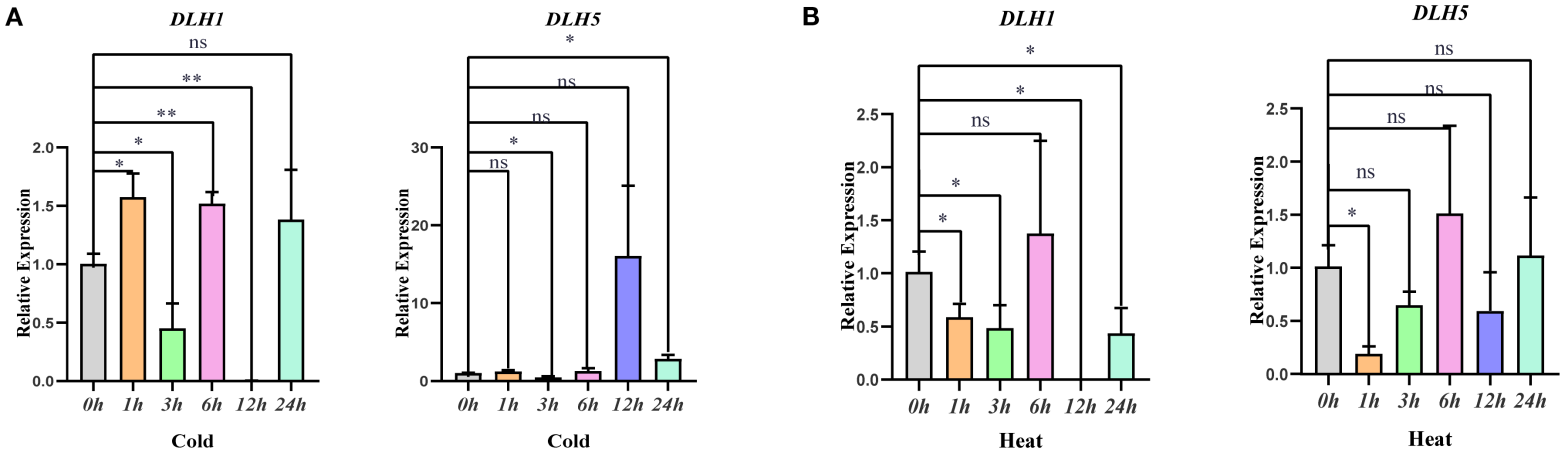
Expression analysis of two genes in the *OsDLH* family under different treatments. **(A)** Cold treatment at 4°C; **(B)** Heat treatment at 42°C. The data were statistically analyzed using WPS 2023 software, and analysis of variance was performed using IBM SPSS Statistics 25 software. The significance levels were defined as *** p < 0.001, ** p < 0.01, * p < 0.05.

Transcriptome data analysis showed that the expression level of the *OsDLH1* gene increased from 111 to 124 after 6 h of cold stress. The expression level of the *OsDLH5* gene increased from 108 to 178 after 12 h of heat stress. Based on *cis-*acting element analysis and the above RNA-seq transcriptome data mining, this study screened two genes, *OsDLH1* and *OsDLH5*, as key research objects and made an in-depth analysis on their expression patterns under different environmental conditions (**Figure 4**). Under cold stress, the expression of these two genes generally showed an upward trend. In particular, the expression level of *OsDLH5* increased significantly at 24 h. Under heat treatment, the expression of these two genes exhibited an overall downward trend despite a peak at 6 h. These results indicated that *OsDLH1* and *OsDLH5* have specific expression patterns in response to different environmental signals, implying that they may play different roles in the growth and development process of rice and the stress response mechanism.

### 3.6 Diversity analysis of *OsDLH* alleles in different rice populations

To understand the potential role of *OsDLH* alleles in rice improvement, we utilized gcHap data from 3KRG to calculate the Shannon equitability (EH) values of 11 *OsDLH* genes, the frequency of gcHaps, and the number of major gcHaps (gcHaN) (with a frequency of ≥1% in 3KRG) in four major rice populations (**Supplementary Table S3**). Among the 11 *OsDLH* genes, the average number of gcHaps, the average number of major gcHaps, and the EH value were 349.5, 11.3, and 0.381, respectively, indicating differences in their genetic diversity. In particular, *OsDLH10* had the highest EH value (0.595), while *OsDLH2* had the lowest EH value (0.129). This difference corresponded to their respective number of gcHaps, with *OsDLH10* having 738 gcHaps, while *OsDLH2* having only 50 gcHaps (**Supplementary Table S3**).

Further observation of the genetic diversity among different rice populations revealed that the average EH value of the 11 *OsDLH* genes also had significant differences among various populations. The average EH value of the Xian, Geng, Aus, Bas, and Mixed populations was 0.43, 0.33, 0.421, 0.516, and 0.659 (the highest), respectively. There were also great variations in the average number of detected gcHaps and major gcHaps, which were 246 and 12.7 for the Xian population, 82.7 and 6.8 for the Geng population, 38.8 and 8.7 for the Aus population, 23.5 and 4.3 for the Bas population, and 44.5 and 10.8 for the Mixed population, respectively (**Supplementary Table S3**).

The F-statistic shows that the Fst values of the 11 DLH genes range from 0.0371 (DLH4) to 0.3521 (DLH2). There is a gradient distribution of the degree of differentiation among the five rice populations, namely Xian, Geng, Aus, Bas, and admix. Among them, DLH3 and DLH4 show low differentiation, DLH6, DLH7, DLH10, DLH1, DLH8, DLH9, and DLH11 exhibit moderate differentiation, while DLH2 and DLH5 show a relatively high degree of differentiation(**Supplementary Table S3**).

**Figure 5:**
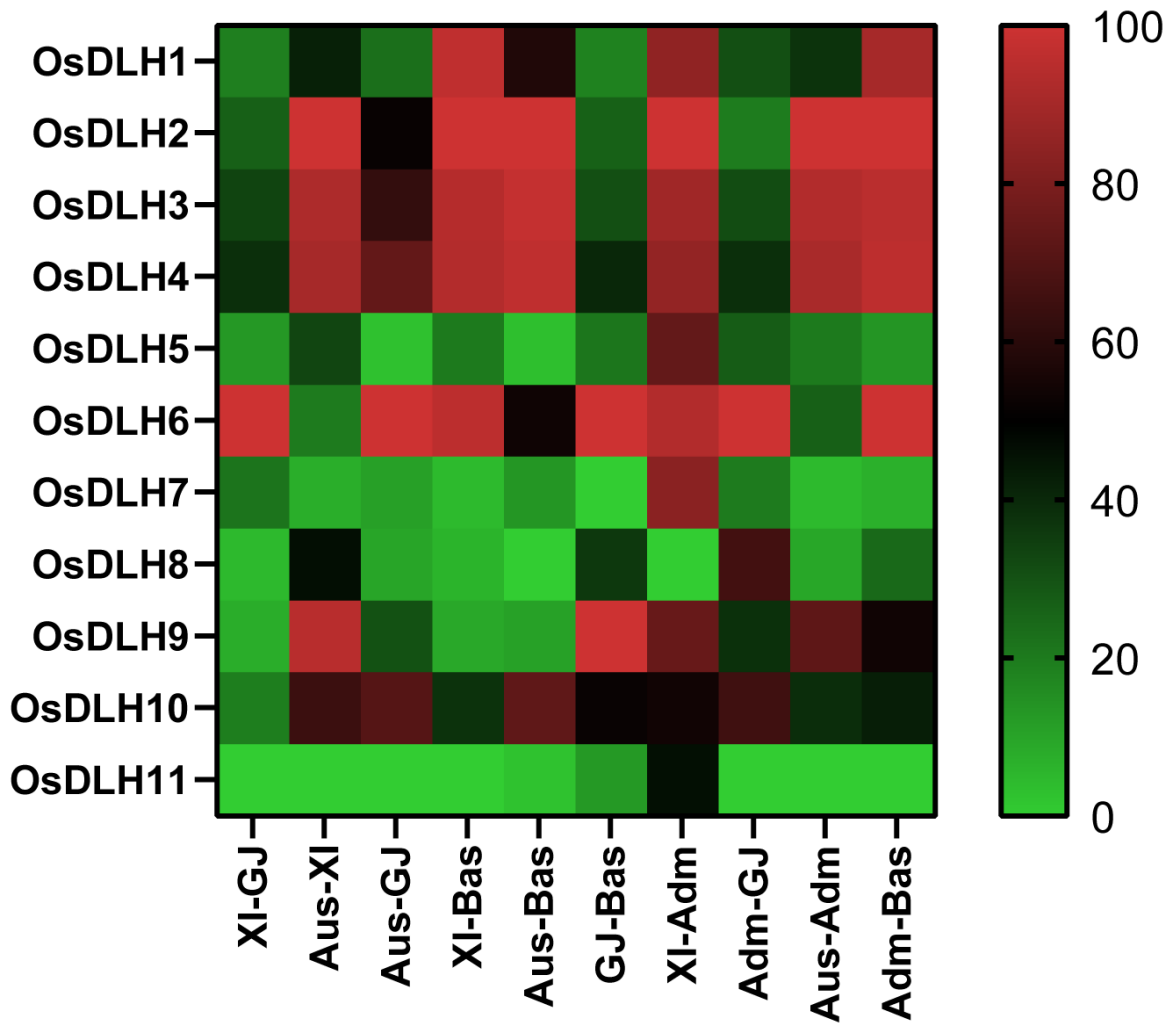
Genetic diversity index (INei). Pairwise comparisons were carried out for different populations calculated from gcHap data.

To understand the genetic differences in *OsDLH* genes among major rice populations, we analyzed the gcHap data of 11 *OsDLH* genes between all pairwise populations using the genetic diversity index (INei). The results showed that *OsDLH7, OsDLH8, OsDLH9*, and *OsDLH11* exhibited significant genetic differentiation (INei < 0.35) in the Aus-Bas, XI-Bas, and Aus-GJ pairwise comparisons (**Figure 5**, **Supplementary Table S4**). *OsDLH11* also showed strong genetic differentiation in the XI-GJ, Aus-GJ, XI-Bas, Aus-Bas, and GJ - Bas pairwise comparisons. These results suggested that allelic variations at the *OsDLH* loci significantly contribute to the differentiation of rice populations and their adaptation to different environments.

### 3.7 Influence of modern breeding on the gcHap diversity of *OsDLHs*

To investigate the impact of modern breeding on the gcHap diversity in *OsDLH* genes in recent decades, we compared their genetic diversity in modern varieties (MVs) and landraces (LANs). The comparison included 732 landraces (LANs-Xian) and 358 modern varieties (MVs-Xian) in the Xian population, as well as 328 landraces (LANs-Geng) and 139 modern varieties (MVs-Geng) in the Geng population (**Tables 1**, 2). In the Xian population, MVs-Xian (0.549) had a higher average EH value of the 11 *OsDLH* genes than LANs-Xian (0.108), and almost all *OsDLH* genes in MVs-Xian had a higher genetic diversity than those in LANs-Xian. However, it is noteworthy that the average value of gcHapsN in MVs-Xian was 36.8 lower than that in LANs-Xian. The increase in genetic diversity while the decrease in gcHapsN observed at multiple *OsDLH* loci in MVs-Xian reflect a significant change in the frequency of gcHaps at these loci during the modern breeding process. In fact, except for *OsDLH5* and *OsDLH9*, the number of gcHaps at other nine *DLH* gene loci decreased significantly in MVs-Xian (**Table 1**).

**Table 1:** Comparison of genetic diversity of 11 *OsDLH* genes between *indica* landraces and modern varieties.

Genename	Xian(indica)							
	LANs		MVs		MVs-LANs		statistical significance	Artificial selection effect
	E*_H_*	gcHapN	E*_H_*	gcHapN	E*_H_*	gcHapN		
OsDLH1	0.596	173	0.711	129	0.115	-44	**	up
OsDLH2	0.070	19	0.110	16	0.040	-3	*	up
OsDLH3	0.642	248	0.658	121	0.016	-127	ns	down
OsDLH4	0.530	156	0.595	99	0.065	-57	*	up
OsDLH5	0.224	9	0.274	10	0.050	1	ns	down
OsDLH6	0.444	117	0.581	82	0.137	-35	**	up
OsDLH7	0.399	53	0.525	45	0.126	-8	**	up
OsDLH8	0.347	77.0	0.488	71.0	0.141	-6	**	up
OsDLH9	0.384	91.0	0.597	91.0	0.213	0	**	up
OsDLH10	0.622	236.0	0.768	155.0	0.146	-81	**	up
OsDLH11	0.589	177.0	0.730	132.0	0.141	-45	**	up
Mean	0.441	123.3	0.549	86.5	0.108	-36.81818182		

EH and gcHapN (major gcHapN) refer to Shannon’s equitability and the number of identified gcHaps (in varieties with ≥ 1% frequency), ** p<0.0001.

In addition, in the Geng population, the average EH of 11 *OsDLH* genes in MVs-Geng and LANs-Geng was 0.317 and 0.311, respectively. In particular, MVs-Geng showed significant increases in genetic diversity only at the *OsDLH3* and *OsDLH9* loci. The average value of gcHapsN in MVs-Geng was 23.5, which is 22.4 lower than that in landraces (**Table 2**). In addition, the dominant gcHap Hap1 and gcHap Hap2 were observed at all *OsDLH* loci in both the Xian and Geng populations, indicating their important roles in these two populations (**Supplementary Table S5**).

**Table 2:** Comparison of genetic diversity of 11 *OsDLH* genes between Geng landraces and modern varieties.

Gene name	*Geng (japonica)*							
	LANs		MVs		MVs-LANs		Statistical significance	Artificial selection effect
	*E_H_*	gcHapN	*E_H_*	gcHapN	*E_H_*	gcHapN		
OsDLH1	0.250	46	0.268	28	0.018	-18	ns	down
OsDLH2	0.154	18	0.186	11	0.032	-7	ns	down
OsDLH3	0.538	98	0.464	38	-0.074	-60	*	up
OsDLH4	0.501	87	0.464	34	-0.037	-53	ns	down
OsDLH5	0.041	5	0.062	6	0.021	1	ns	down
OsDLH6	0.424	73	0.364	25	-0.060	-48	ns	down
OsDLH7	0.318	19	0.343	15	0.025	-4	ns	down
OsDLH8	0.203	32.0	0.155	15.0	-0.048	-17	ns	down
OsDLH9	0.316	28.0	0.438	24.0	0.122	-4	**	up
OsDLH10	0.433	59.0	0.456	38.0	0.023	-21	ns	down
OsDLH11	0.244	40.0	0.283	25.0	0.039	-15	ns	down
Mean	0.311	45.9	0.317	23.5	0.006	-22.4		

EH and gcHapN (major gcHapN) represent Shannon’s evenness and the number of identified gcHaps (in varieties accounting for ≥ 1%), ** p<0.0001.

### 3.8 Comparison of the trait values of favorable and unfavorable gcHaps of *OsDLHs*

The dominant gcHaps at the *OsDLH* loci in rice, namely those with the highest frequency, are considered to be favored by natural selection during the evolution process. On the contrary, the main gcHaps with the lowest frequency are probably unfavorable gcHaps (Zeng et al., 2023). We compared the phenotypic differences in 15 agronomic traits between the favorable and unfavorable gcHaps at eight *OsDLH* loci (**Supplementary Table S6**). In a total of 120 comparisons, differences were detected in 68 comparisons (**Supplementary Figure S1-S8**). Among the eight *OsDLH* genes, for the 1000-grain weight (TGW), phenotypic differences were observed between favorable and unfavorable gcHaps in five genes. Notably, *OsDLH2* showed the greatest differences, with phenotypic differences between favorable and unfavorable gcHaps in all the 15 traits.

### 3.9 Relationship between the main gcHaps of *OsDLHs* and important agronomic traits

**Figure 6:**
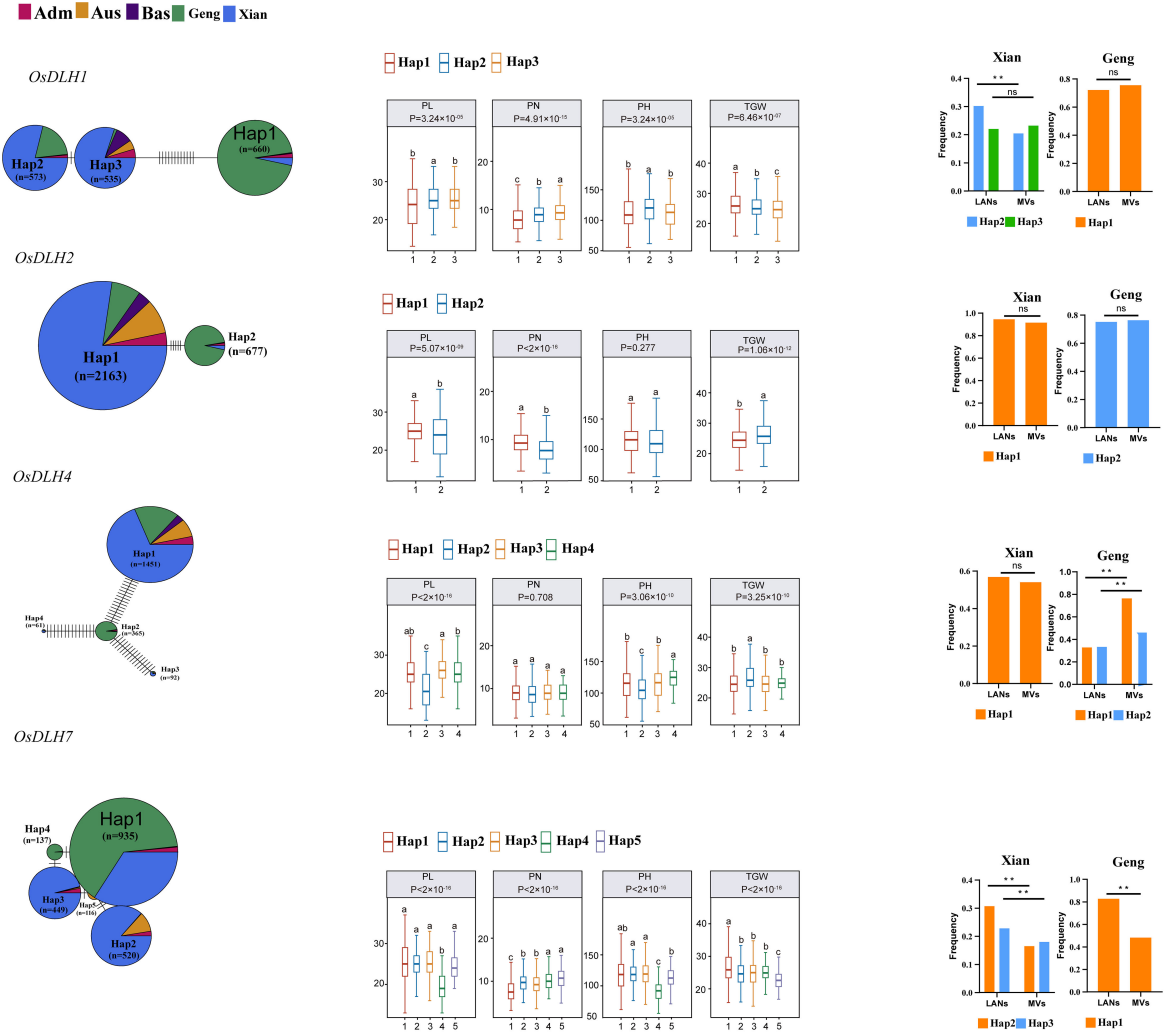
Haplotype networks of four cloned *OsDLH* genes and four agronomic traits in 3KRG. Letters denote differences among haplotypes evaluated by two-way analysis of variance, where different letters on the box and whisker plots indicate statistically significant differences at p < 0.05 according to Duncan’s multiple-range test. The bar chart on the right shows the frequency differences of major gcHaps between landraces (LANs) and modern varieties (MVs) of Xian and Geng. The chi-square test was used to determine significant differences in the proportion of gcHaps among different populations (** p < 0.0001).

Among the 11 *OsDLHs*, three genes had only one gcHap in 3KRG, and therefore cannot be analyzed for gcHap diversity. To demonstrate the functional importance of *OsDLHs*, we constructed gcHap network of the dominant alleles of the remaining genes in 3KRG in five rice populations, and analyzed the relationships between them and four agronomic traits, namely panicle number per plant (PN), panicle length (PL), plant height (PH), and TGW (**Figure 6**; **Supplementary Figure S9**). Strong correlations (P < 10^-7^) were observed in 23 out of the 32 (8 × 4) analyzed cases, and the major alleles of multiple *OsDLH* genes were significantly associated with the values of one or more traits. *OsDLH1* had three major gcHaps, with Hap2 having a relatively high frequency in LANs-Xian. Compared with Hap1 and Hap3, Hap2 significantly increased PL and PH during the breeding process. *OsDLH2* had two major gcHaps, with Hap1 having a relatively high frequency in LANs-Xian. Compared with Hap2, Hap1 significantly increased PL and PN during the breeding process. *OsDLH4* had four major gcHaps, where Hap1 had a relatively high frequency in both LANs-Xian and MVs-Xian. Compared with Hap2, Hap1 also significantly increased PL and PH during the breeding process. *OsDLH9* had five major gcHaps, with Hap1 having a relatively high frequency in both LANs-Geng and MVs-Geng. Compared with Hap2 and Hap3, Hap1 significantly increased TGW during the breeding process.

## 4 Discussion

Cellulose is the predominant biopolymer and the core structural component within the plant cell wall (Etale et al., 2023; Lampugnani et al., 2019). The biosynthesis of cellulose in plant cell walls by CSCs is controlled by a sophisticated regulatory framework (Li et al., 2014b; Polko and Kieber, 2019; Pedersen et al., 2023). Although the functions of some *OsDLH* genes have been reported in barley and sorghum, their regulatory mechanisms remain largely unexplored, with only a few cases investigated. For example, barley (1,3;1,4)-β-d-glucanase is believed to have evolved from an ancestral monocotyledon (1,3)-β-d-glucanase, enabling the hydrolysis of (1,3;1,4)-β-d-glucans in the cell walls of leaves and germinating grains (Kao et al., 2024). The changes in enzyme activities in sorghum malt showed that the activity of endo-(1,3)(1,4)-β-d -glucanase was lower than that of α-amylase (Uriyo and Eigel, 2000). Here, we identified the members of the *OsDLH* family and investigated their functions by gene expression and evolutionary tree (**Figures 1** and **3**). With the publication of the TFsβ-glucanase-β-1,4-1,3-cellotriose complex structure, it has been revealed how the product β-1,4-1,3-cellotriose binds to the enzyme active site (Tsai et al., 2005). Structural modeling of glucanase - substrate complexes indicated that the tyrosine residue, conserved across all known 1,3-1,4-β-D-glucanases, plays a role in the recognition of polysaccharides with mixed β-1,3 and β-1,4 linkages (Tsai et al., 2008). This specific structural feature provides important reference for the identification of the *DLH* gene family in cereals.

Analyses of RNA-seq data (**Figure 3**) and qPCR results (**Figure 4**) of the *OsDLH* gene family indicated that different *OsDLH* genes have specific expression responses to environmental signals and stress conditions such as ABA, MeJA, drought, heat, cold, and salt stress. *OsDLH1* showed relatively high expression levels under multiple stress conditions, while *OsDLH10* exhibited a unique expression pattern under drought and salt treatments. The expression of *OsDLH2* and *OsDLH8* was up-regulated under hormone treatments and was quickly responsive to salt stress. This result is consistent with the findings of Tamoi. They found that SsGlc is a novel type of GH9 glucanase, which could specifically hydrolyse the β-1,3-1,4-linkage of glucan. However, under salt stress (300–450 mM NaCl), the growth of the Ssglc-disrupted mutant cells was significantly inhibited as compared with that of the wild-type cells (Tamoi et al., 2007). *DLH* genes are involved in the synthesis and decomposition of cellulose. Abnormalities in *DLH* genes can lead to abnormal cell wall synthesis, thus reducing plant resistance to stress. The expression patterns of these genes are closely related to their biological functions, but the specific mechanisms still require further investigation.

Analysis of the gcHap data from the 3KRG project revealed that the *OsDLH* gene family has significant genetic diversity among different populations, particularly the admixed population. Genetic differentiation analysis indicated that *OsDLH1, OsDLH2*, and *OsDLH10* had significant differences in genetic variation among different rice populations, which may have important contribution to rice adaptability and population differentiation. Comparison between modern varieties and landraces showed that although modern Xian varieties have high genetic diversity, they possess a relatively smaller number of gcHaps, probably due to gene recombination. In the Geng population, modern varieties had low genetic diversity at the *OsDLH3* locus, but the main gcHap, Hap1, had a consistent frequency across all loci, indicating its stability in different populations. These results demonstrate that breeding has a profound impact on the genetic diversity of rice, and allelic diversity and environmental adaptability should be considered in the breeding programs.

A comparison of the performance of favorable and unfavorable gcHaps at eight *OsDLH* loci for 15 agronomic traits demonstrated that the two haplotypes of *OsDLH2* had the largest phenotypic differences, indicating that this gene has significant impacts on multiple agronomic traits. Except for those of *OsDLH4* and *OsDLH6*, some specific gcHaps of other *OsDLH* genes were significantly associated with traits such as GW and DTH, highlighting the necessity of considering allelic diversity in rice breeding. An in-depth association analysis showed that the gcHaps of the major alleles of 11 *OsDLH* genes were significantly associated with some agronomic traits such as PN, PL, PH, and TGW in five rice populations, with highly significant associations being observed in 42.2% of the analyzed cases. Our results also revealed that the frequencies of some favorable gcHaps at *OsDLH* loci varied among different rice populations, implying possible adaptive genetic variations in different ecological environments (Zeng et al., 2023). The findings highlight the importance of selecting and utilizing the favorable alleles to improve the allelic diversity and environmental adaptability in rice breeding.

## 5 Conclusion

Phylogenetic and collinearity analyses revealed lineage-specific contraction and differentiation of the *DLH* gene family. *Cis*-element, transcriptome, and qPCR analyses identified *OsDLH1* and *OsDLH5* as the key genes regulating cold and heat tolerance in rice. Integration of multiple datasets constructed a molecular framework for rice stress adaptation, providing targets for improving the climate adaptability of Poaceae. Haplotype 7 of *OsDLH6* and haplotype 1 of *OsDLH9* are haplotypes that significantly affect the seed setting rate. Haplotype differences may help explain the genetic mechanisms underlying their functional divergence.

## Data Availability

The datasets presented in this study can be found in online repositories. The names of the repository/repositories and accession number(s) can be found in the article/**Supplementary Material**.
