# Supplementary material for: Identification of 1,3-1,4-β-D-glucanase (OsDLH) genes and analysis of haplotype diversity in rice
Source: Front Plant Sci. 2025 Oct 16;16:1690795. doi: 10.3389/fpls.2025.1690795 (PMC12571822; doi:10.3389/fpls.2025.1690795)
Supplement: Supplementary file 1 [file DataSheet1.docx]

**Analysis of the Rice 1,3 - 1,4-β-D - glucanase (OsDLH) Gene Family and Haplotype Diversity**


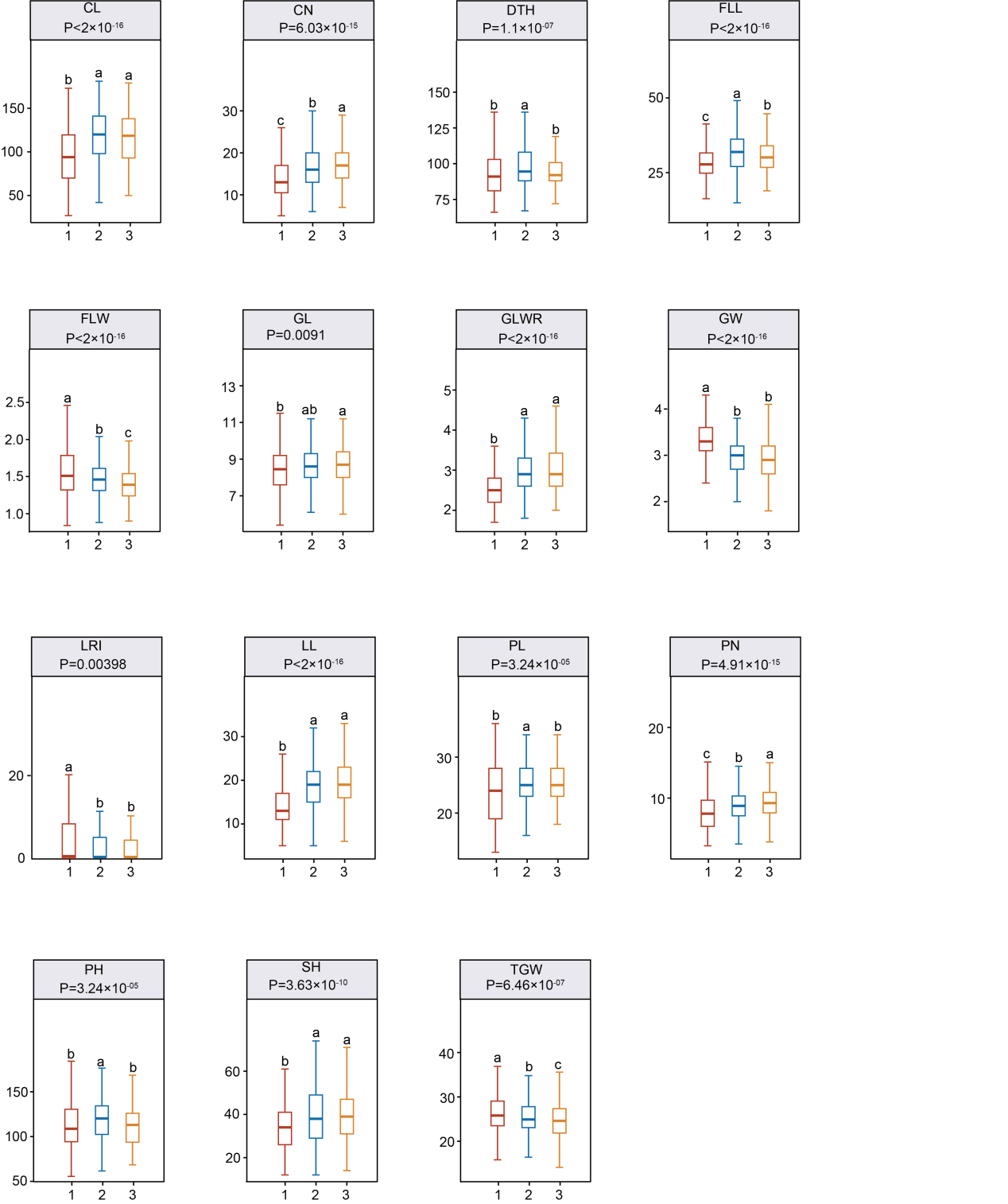


**Figure S1**. Comparison and analysis of 15 agronomic traits among the predominant gcHap, unfavorable

gcHap, and major gcHaps of *OsDLH1.*


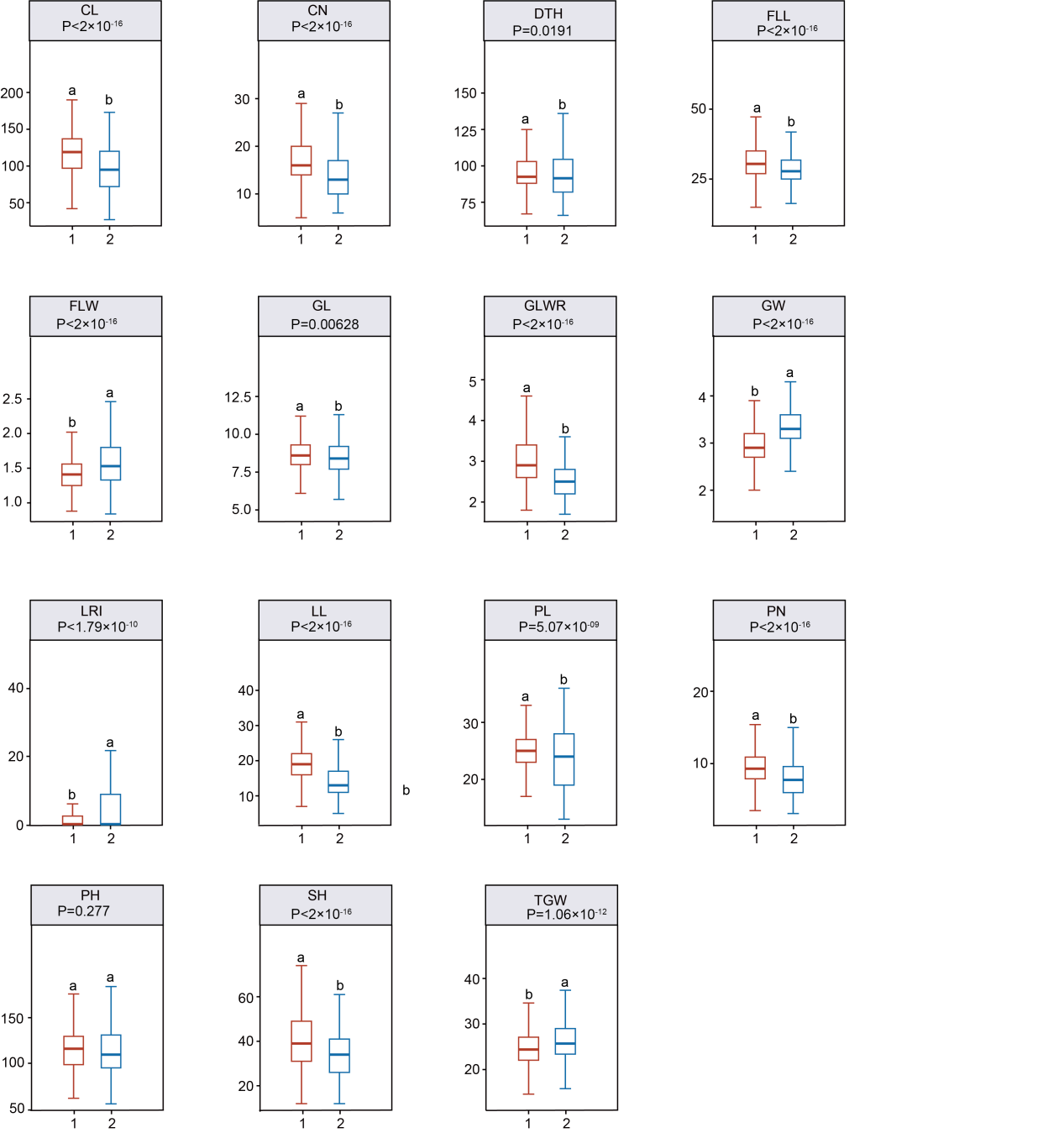


**Figure S2**. Comparison and analysis of 15 agronomic traits among the predominant gcHap, unfavorable

gcHap, and major gcHaps of *OsDLH2.*


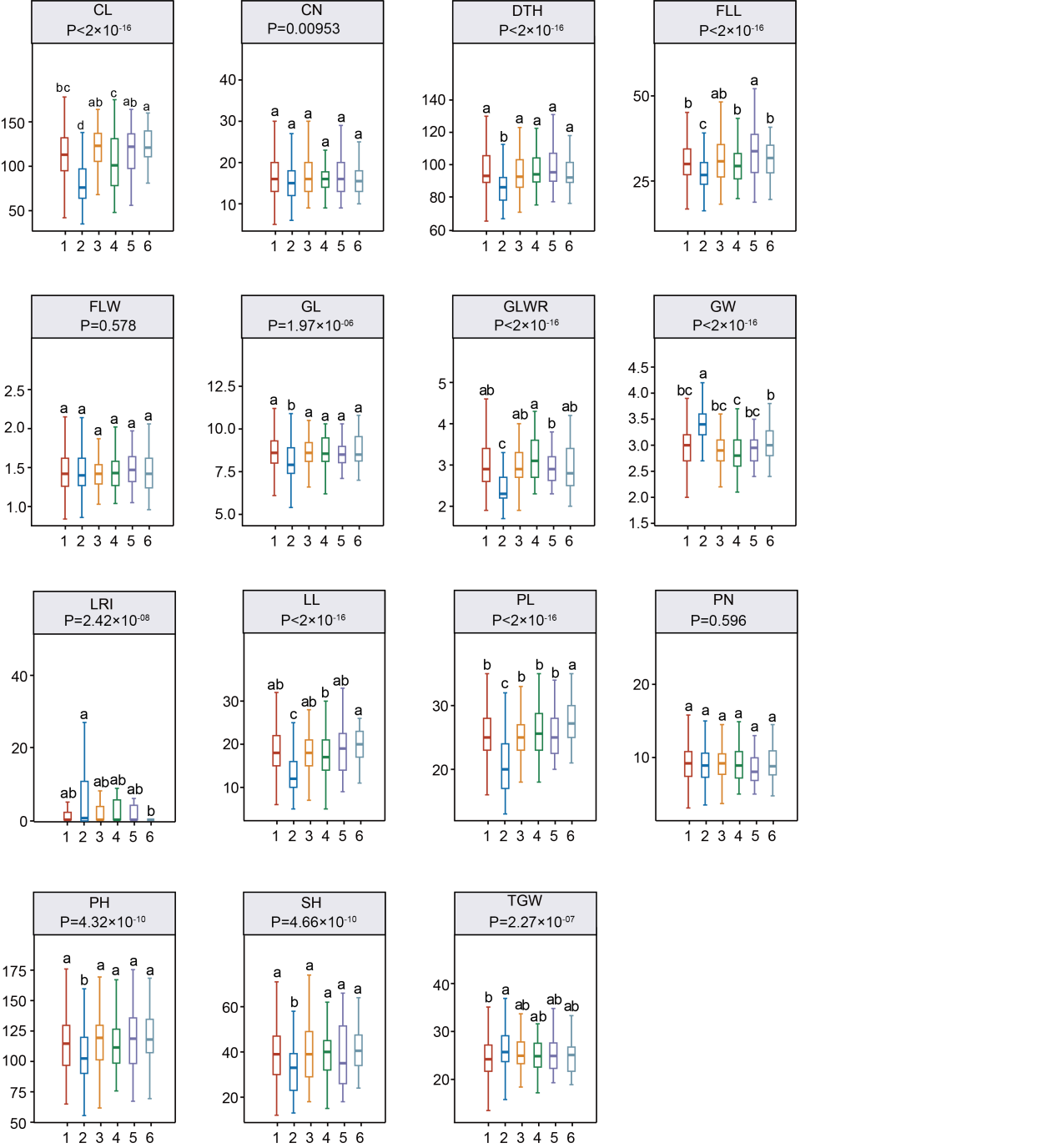


**Figure S3**. Comparison and analysis of 15 agronomic traits among the predominant gcHap, unfavorable

gcHap, and major gcHaps of *OsDLH3.*


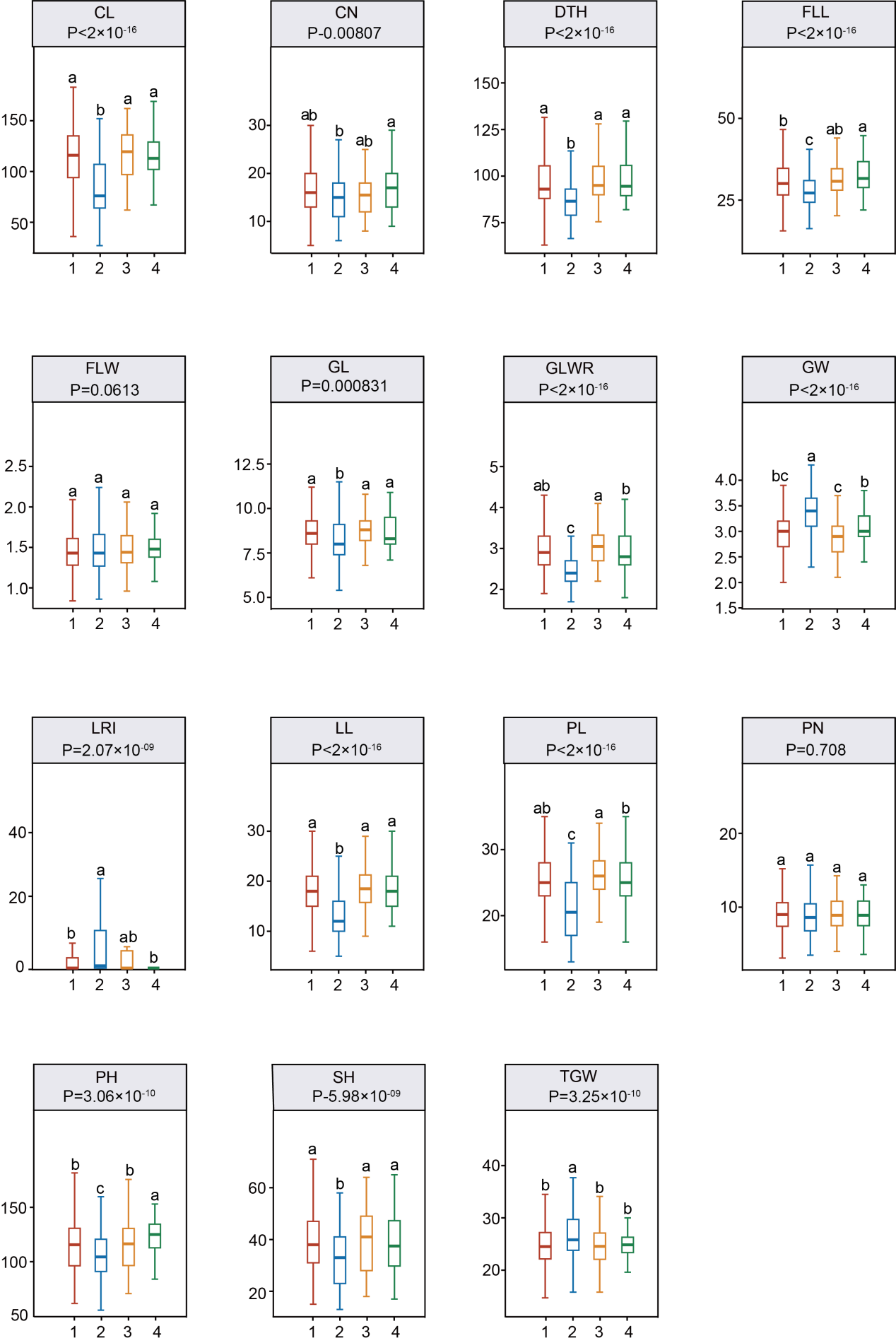


**Figure S4**. Comparison and analysis of 15 agronomic traits among the predominant gcHap, unfavorable

gcHap, and major gcHaps of *OsDLH4.*


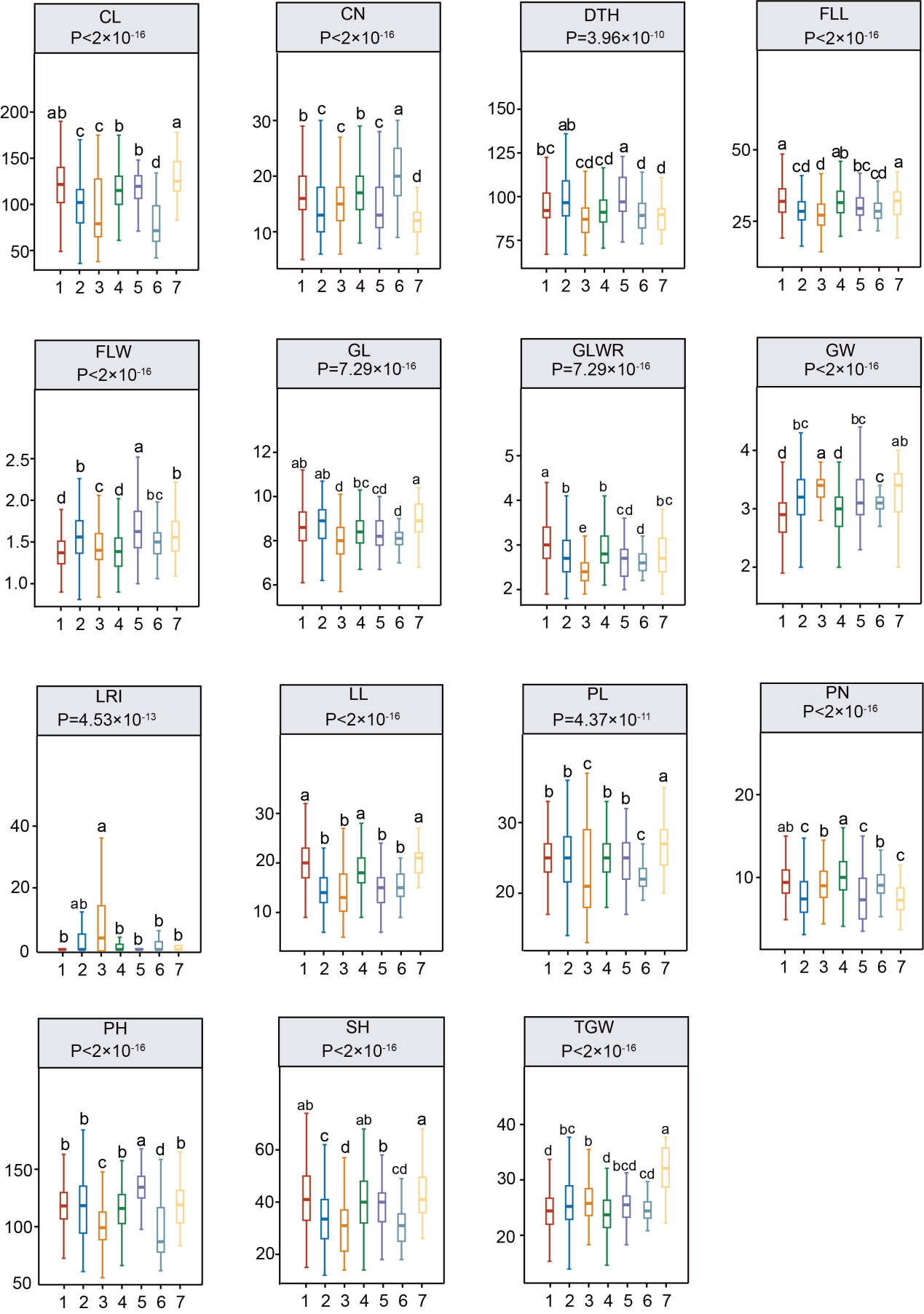


**Figure S5**. Comparison and analysis of 15 agronomic traits among the predominant gcHap, unfavorable

gcHap, and major gcHaps of *OsDLH6.*


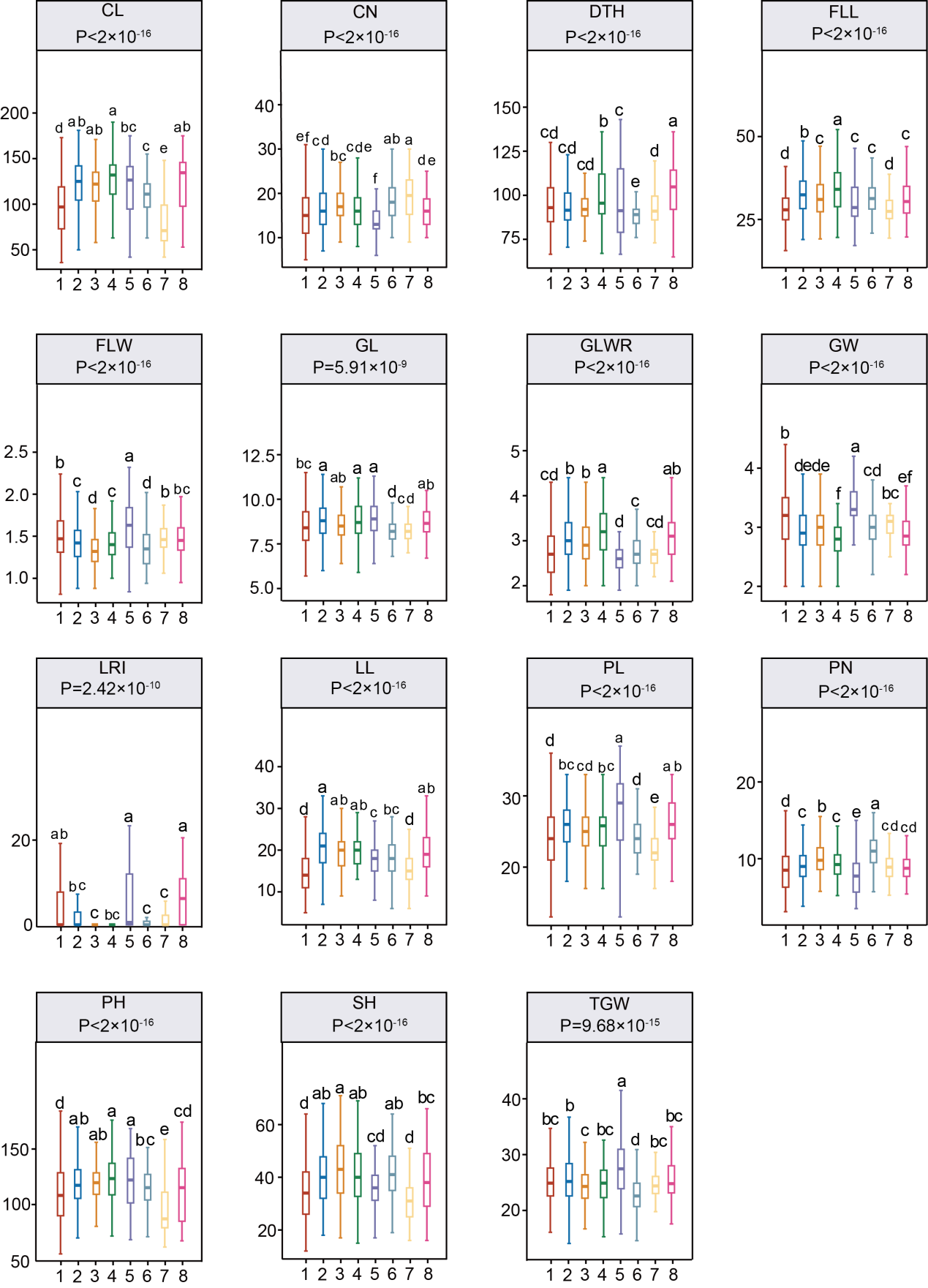


**Figure S6**. Comparison and analysis of 15 agronomic traits among the predominant gcHap, unfavorable

gcHap, and major gcHaps of *OsDLH8*


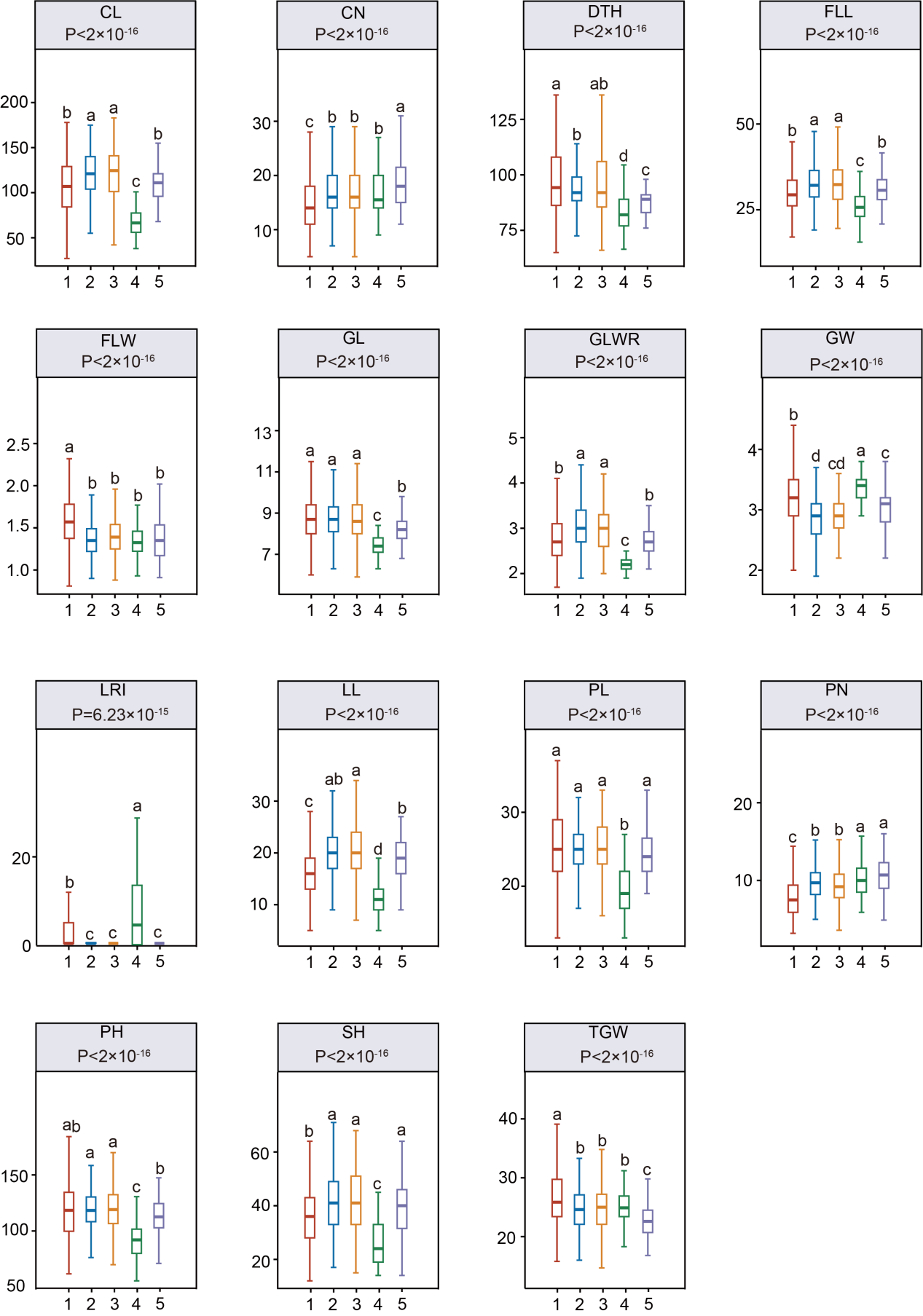


**Figure S7**. Comparison and analysis of 15 agronomic traits among the predominant gcHap, unfavorable

gcHap, and major gcHaps of *OsDLH9.*


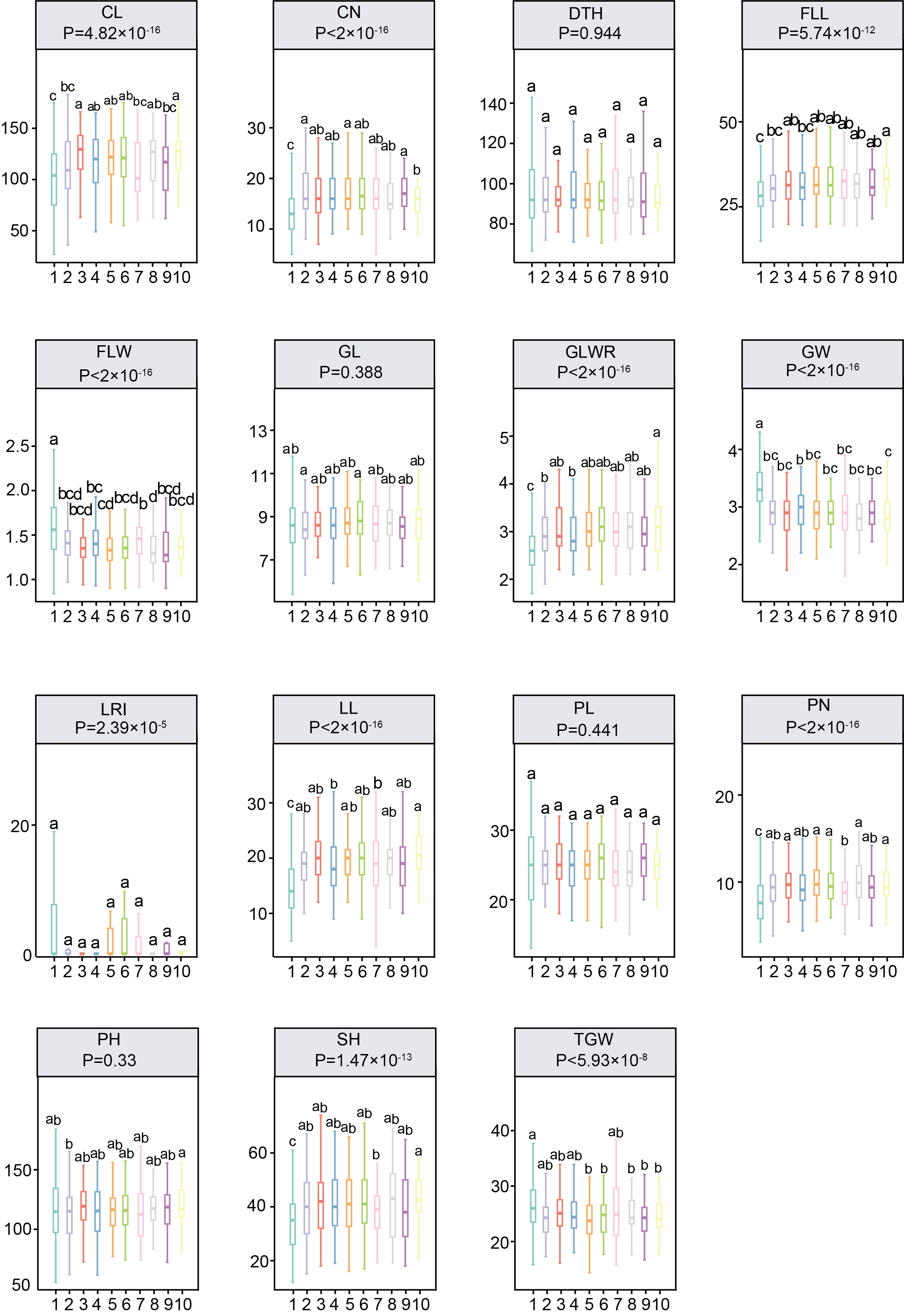


**Figure S8**. Comparison and analysis of 15 agronomic traits among the predominant gcHap, unfavorable

gcHap, and major gcHaps of *OsDLH11.*


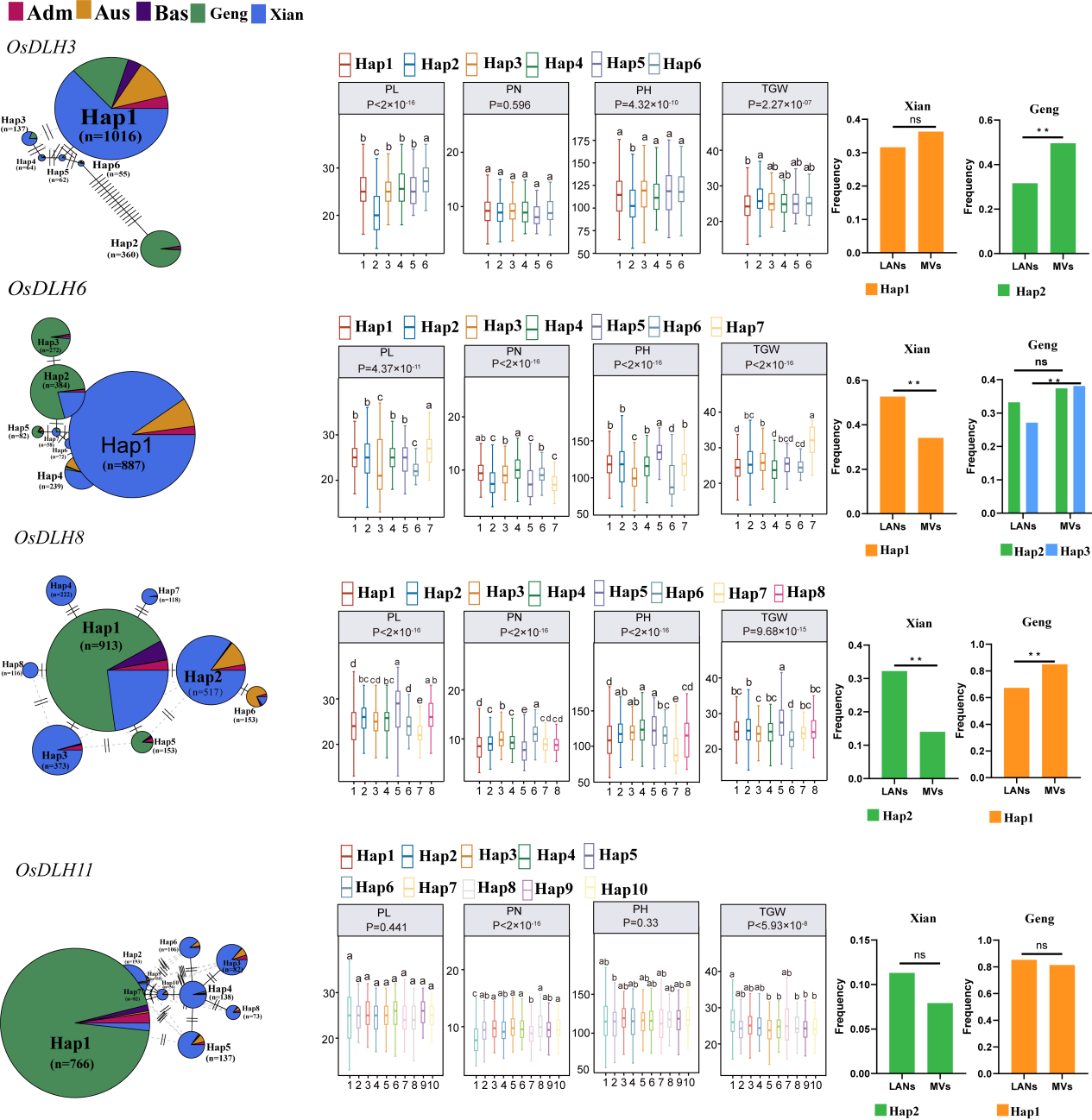


**Figure S9.** Haplotype networks of the four cloned DLH (5~6) genes and their association traits with four

agronomic traits in 3KRG. p-values indicate differences among haplotypes assessed by two-factor

ANOVA, where different letters on the box-and-line plots indicate statistically significant differences

based on the Duncanʹs Multiple Range Test at P<0.05. The bars on the right show the frequency

differences in dominant gcHaps between local varieties (LANs) and modern varieties (MVs) in Xian and

Geng. The chi-square test was used to determine significant differences in the proportions of the same

gcHap between groups ****P<0.0001, ***P<0.001, **P<0.01, *P<0.05 and N.S., not significant.
